# Supplementary material for: Structural basis of differential gene expression at eQTLs loci from high-resolution ensemble models of 3D single-cell chromatin conformations
Source: Bioinformatics. 2025 Jan 31;41(2):btaf050. doi: 10.1093/bioinformatics/btaf050 (PMC11835231; doi:10.1093/bioinformatics/btaf050)
Supplement: btaf050_Supplementary_Data [file btaf050_supplementary_data.docx]

Supplementary Figures


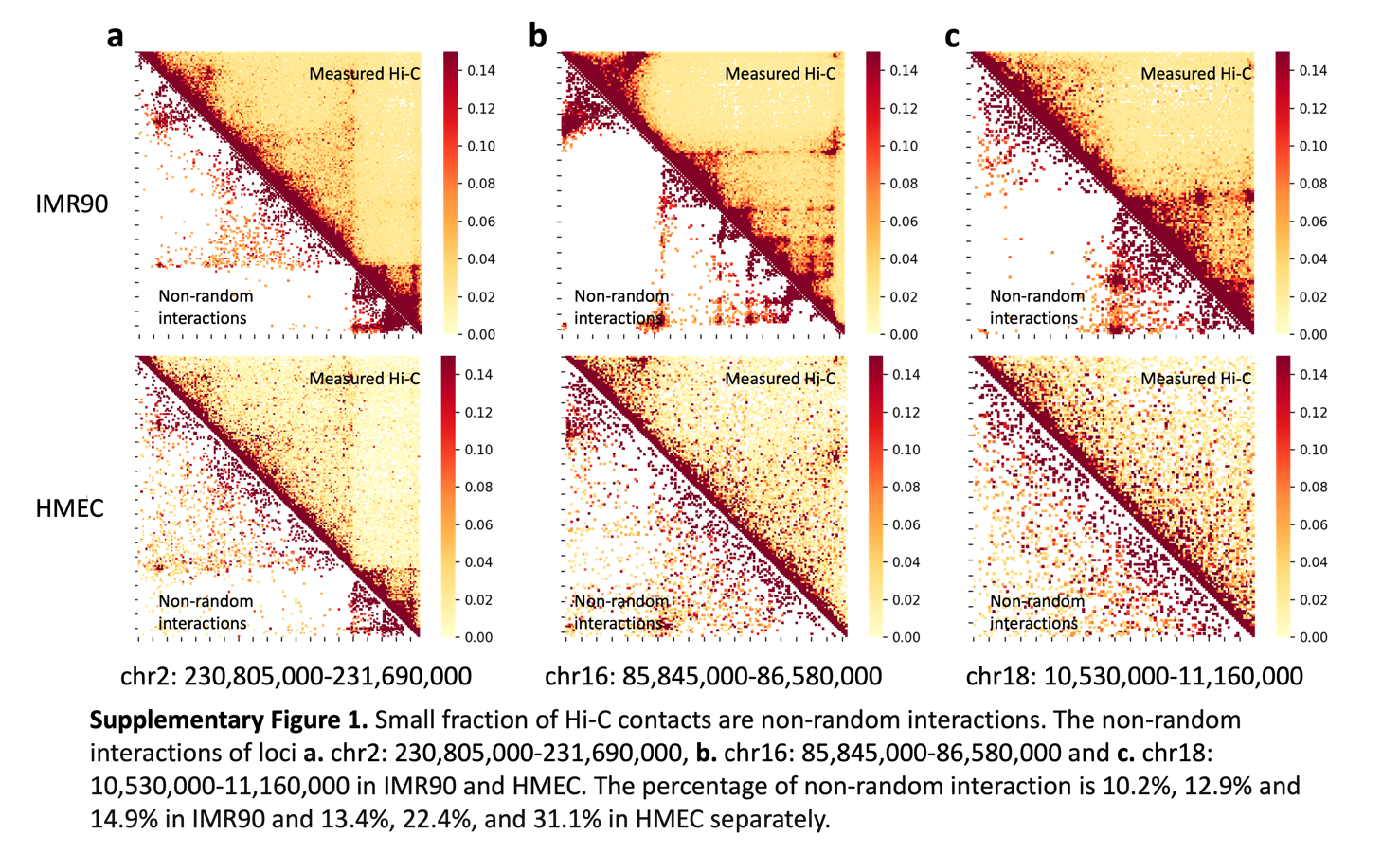


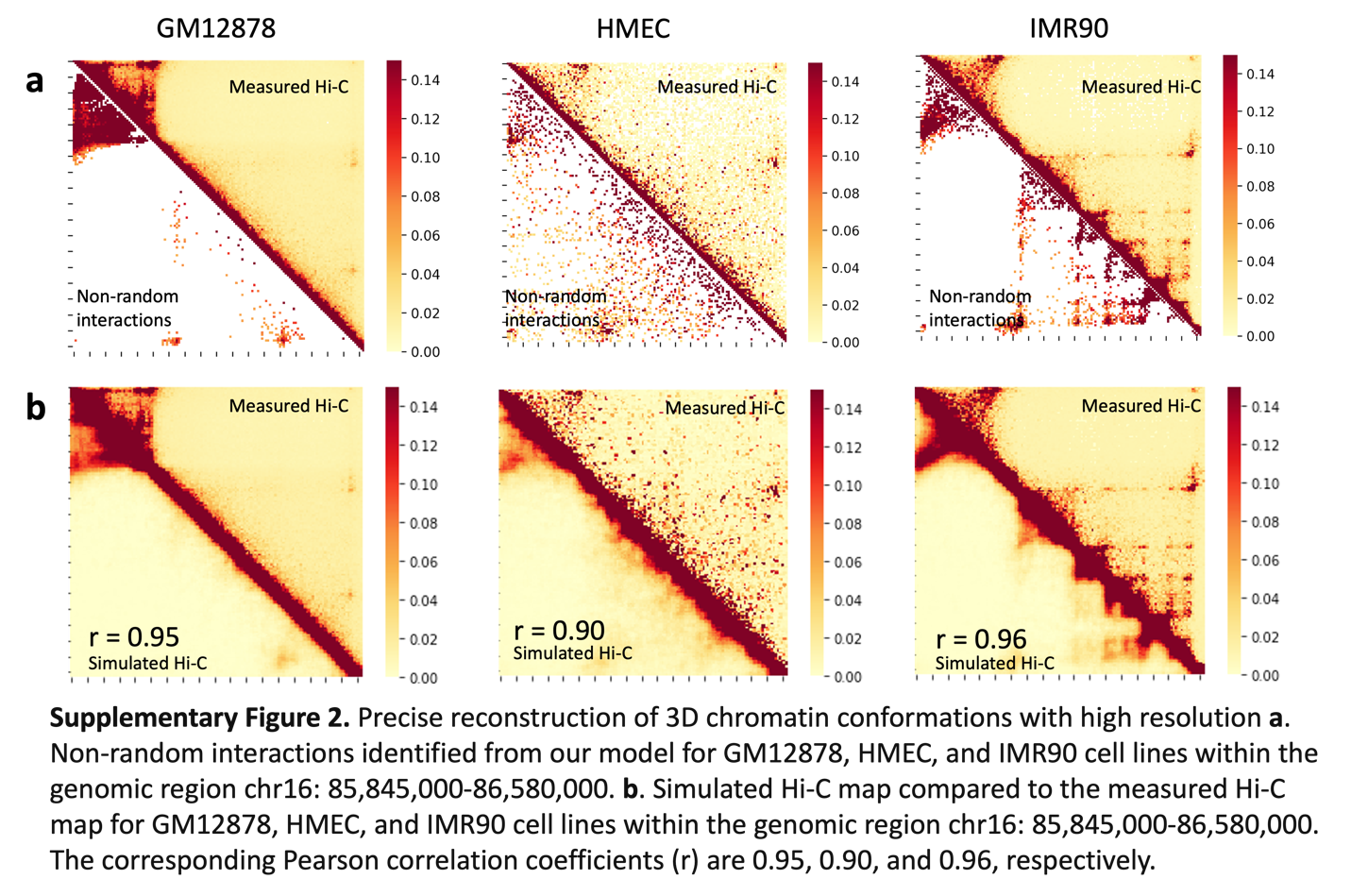


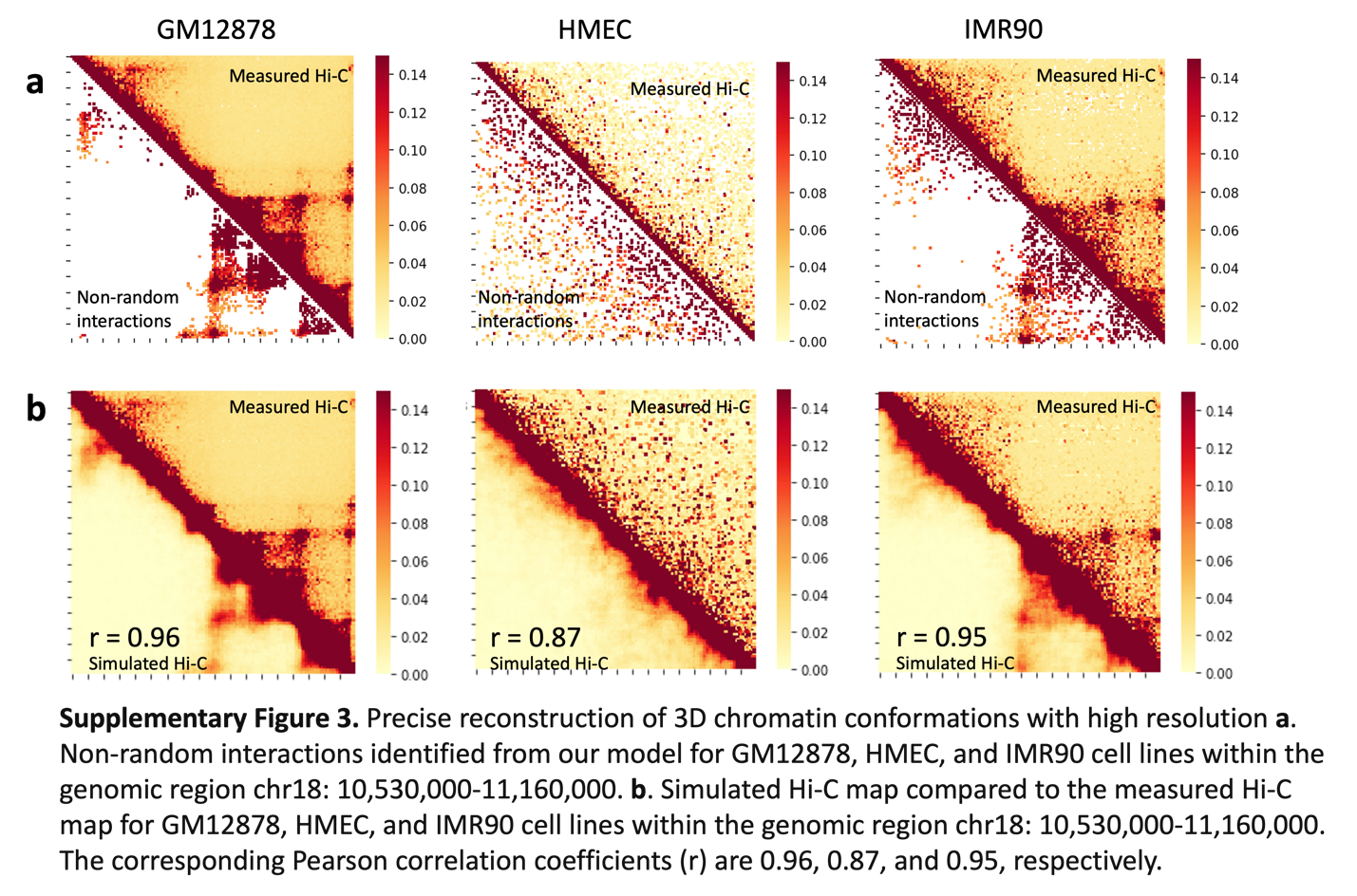


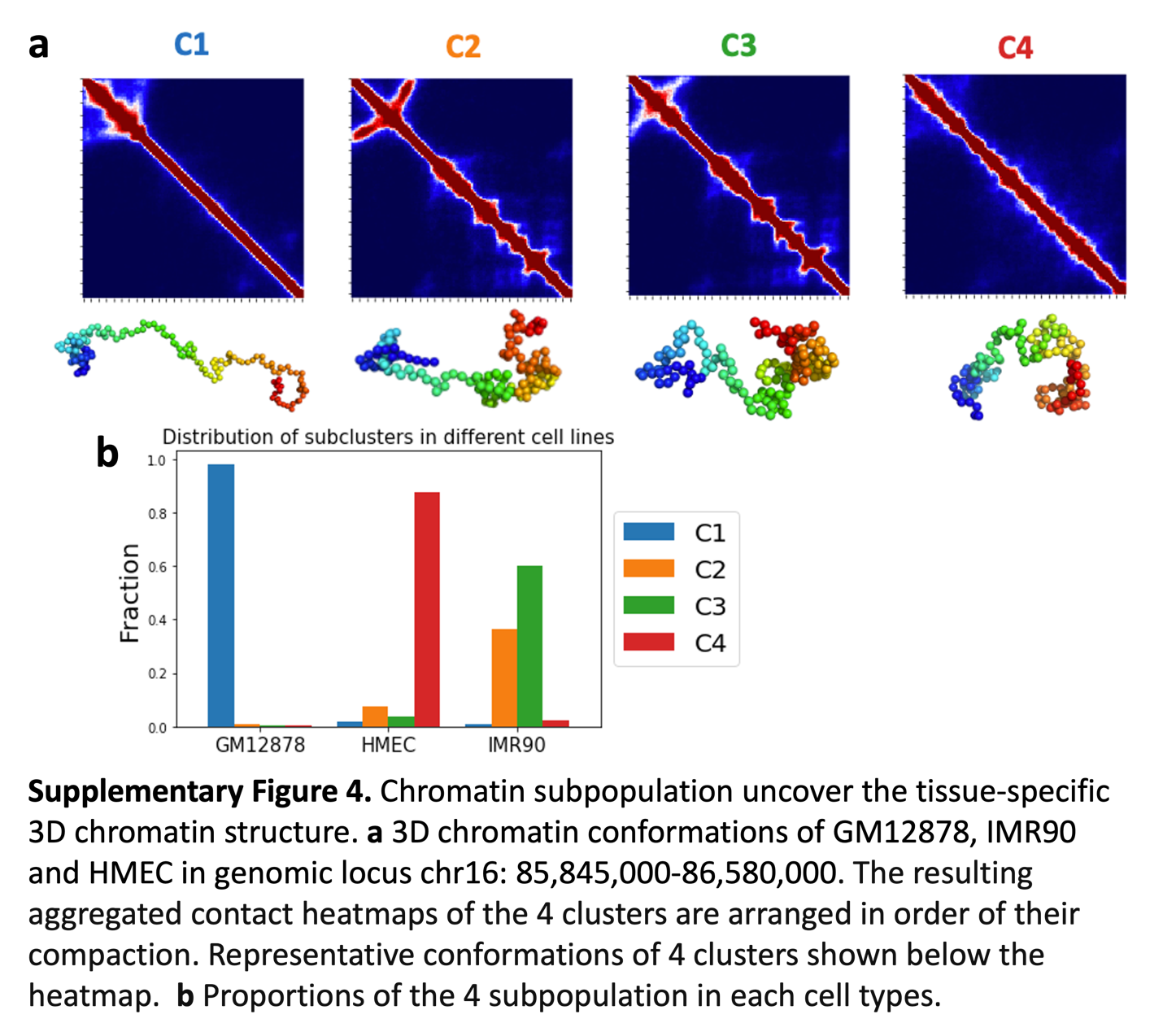


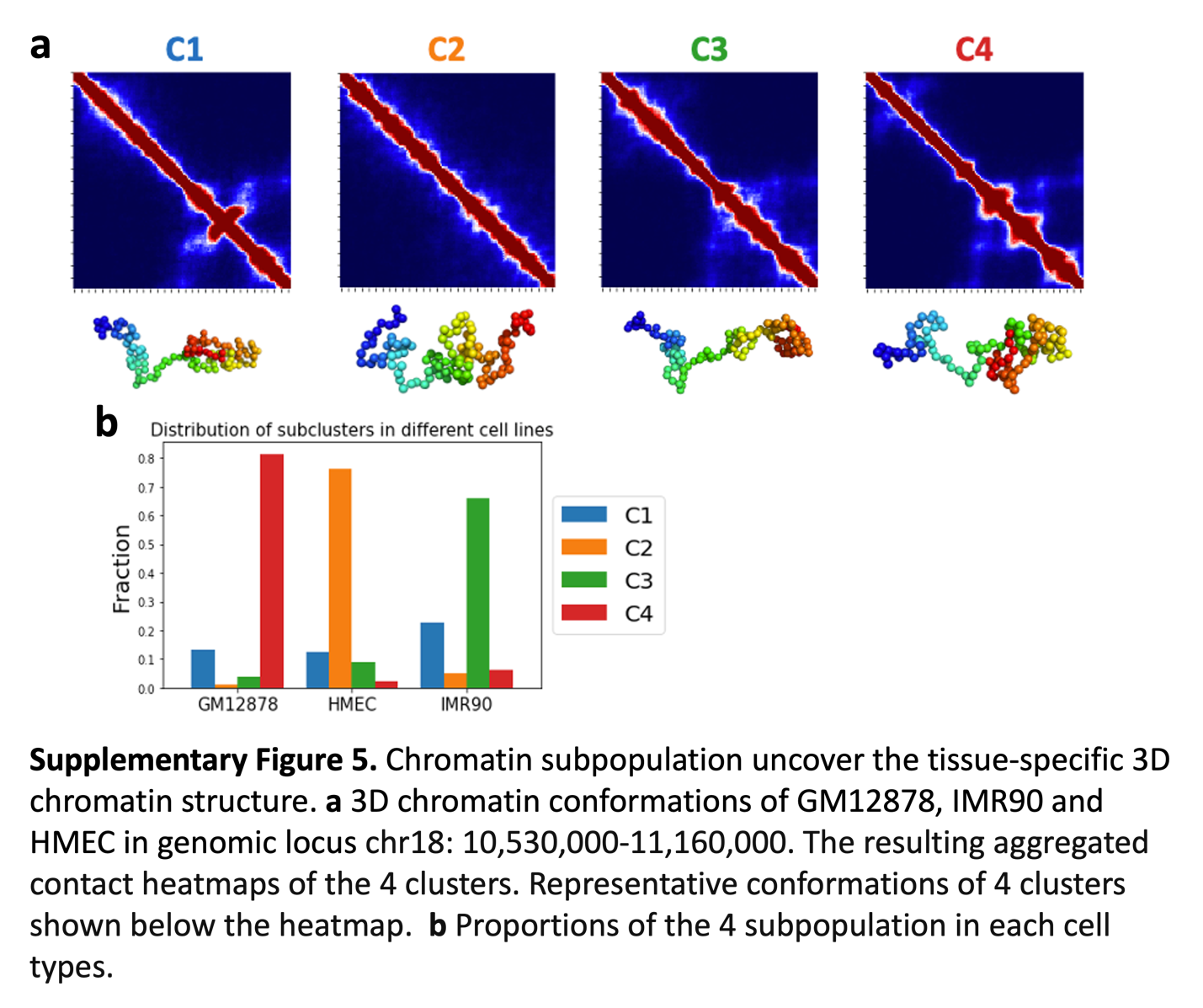


**Table S1.** Correlation coefficients between measured and simulated Hi-C data

|  | GM12878 | | | IMR90 | | | HMEC | | |
| --- | --- | --- | --- | --- | --- | --- | --- | --- | --- |
|  | r^a^ | r_10kb^b^ | r_pl^c^ | r | r_10kb | r_pl | r | r_10kb | r_pl |
| Locus I | 0.97 | 0.96 | 0.45 | 0.96 | 0.94 | 0.45 | 0.94 | 0.87 | 0.46 |
| Locus II | 0.95 | 0.93 | 0.45 | 0.96 | 0.93 | 0.46 | 0.9 | 0.77 | 0.46 |
| Locus III | 0.96 | 0.95 | 0.45 | 0.95 | 0.91 | 0.45 | 0.87 | 0.72 | 0.45 |

a: r is the Pearson correlation coefficient between the measured and our simulated Hi-C data. b: r_10kb is the Pearson correlation coefficient between the measured and our simulated Hi-C after removing proximal interactions (<10 kb) from both measured and simulated Hi-C data. c: r_pl is the Pearson correlation coefficient between the measured and power law based on 1D distance method predicted Hi-C data.
